# Hip Structure Analyses in Acromegaly: Decrease of Cortical Bone Thickness After Treatment: A Longitudinal Cohort Study

**DOI:** 10.1002/jbm4.10240

**Published:** 2019-10-23

**Authors:** Kristin Godang, Tove Lekva, Kjersti Ringvoll Normann, Nicoleta Cristina Olarescu, Kristin Astrid Berland Øystese, Anders Kolnes, Thor Ueland, Jens Bollerslev, Ansgar Heck

**Affiliations:** ^1^ Section of Specialized Endocrinology Oslo University Hospital Oslo Norway; ^2^ Research Institute of Internal Medicine Oslo University Hospital Oslo Norway; ^3^ Faculty of Medicine University of Oslo Oslo Norway; ^4^ KG Jebsen TREC University of Tromsø Tromsø Norway

**Keywords:** ACROMEGALYCORTICAL BONE THICKNESS, DXA, GH/IGF‐1, HIP STRUCTURAL ANALYSIS

## Abstract

Long‐standing growth hormone (GH) excess causes the skeletal clinical signs of acromegaly with typical changes in bone geometry, including increased cortical bone thickness (CBT). However, a high prevalence and incidence of vertebral fractures has been reported. The aim of this study was to assess the course of cortical bone dimensions in the hip by comparing patients with acromegaly and clinically nonfunctioning pituitary adenomas (NFPAs) at baseline and 1 year after pituitary surgery (1‐year PO) in a longitudinal cohort study. DXA was performed in patients with acromegaly (*n* = 56) and NFPA (*n* = 47). CBT in the femoral neck (CBTneck), calcar (CBTcalcar), and shaft (CBTshaft) were determined by hip structural analysis (HSA). CBT at baseline and the change to 1‐year PO were compared. Test results were adjusted for differences in gender distribution, age, and gonadal status. Cortical thickness analyses showed higher values [mm] at baseline in patients with acromegaly compared with NFPA: CBTneck median [25th; 75th] 6.2 [4.7; 8.0] versus 5.1 [4.1; 6.4] (*p* = 0.006), CBTcalcar 4.8 [4.2, 5.7] versus 4.0 [3.2, 4.5] (*p* < 0.001), CBTshaft 6.2 [5.1, 7.2] versus 5.2 [4.6, 6.0], (*p* = 0.003). In acromegaly, GH was correlated with CBTneck (*r* = 0.31, *p* = 0.020), whereas IGF‐1 was correlated with CBTcalcar (*r* = 0.39, *p* = 0.003) at baseline. In acromegaly, CBTneck decreased by 11.2%, *p* = 0.002 during follow‐up. Finally, the decrease in CBTneck and CBTcalcar in acromegaly was significant compared with NFPA (*p* = 0.023 and *p* = 0.017, respectively). Previous observations of increased CBT in acromegaly were confirmed with DXA‐derived HSA in a large, well‐defined cohort. The decline in CBT in acromegaly could contribute to the increased fracture risk in acromegaly despite increased bone dimensions and disease control. © 2019 The Authors. *JBMR Plus* published by Wiley Periodicals, Inc. on behalf of American Society for Bone and Mineral Research.

## Introduction

The typical skeletal features of acromegaly are pathognomonic for the disease as coarsened, enlarged facial appearance, frontal bossing, enlarged jaw, broadened hands and feet, and kyphosis. These changes affect the patient's appearance and also bone health, leading to a surprisingly increased fracture risk despite continuous growth hormone‐ (GH‐) mediated stimulation of bone growth and turnover.^1^, ^2^ GH plays an important role in maintaining bone mass in normal adults by regulating circulating and locally produced insulin‐like growth factors (IGFs) that stimulate bone remodeling.^3^, ^4^ However, though the excess GH/IGF‐1 correlates with increased bone mass in regions with predominantly cortical bone such as the femoral neck,^5^, ^6^, ^7^, ^8^ biomechanical competence of trabecular bone, which dominates in the spine, is reduced in active acromegaly.^9^, ^10^ Thus, patients with newly diagnosed acromegaly have an increased risk of vertebral fractures, but low risk for peripheral fractures,^11^ potentially because of increased CBT as assessed by pQCT.^12^

The different mechanical demands to bone in a certain region determine the predominance of either trabecular or cortical bone. Vertebral bodies must resist high and repetitive axial compression loads and if the trabecular bone is reduced, the stress on the cortical compartments increases and the vertebral bone's ability to resist compression forces decreases.^13^ In contrast, the femoral neck and proximal hip are exposed to shear forces and bending moments, and therefore have distinct cortical structures. The increased risk for vertebral fractures in acromegaly does persist even after treatment^1^, ^14^; accordingly, we have recently demonstrated a decrease in trabecular bone score (TBS) following treatment in our large cohort of prospectively followed patients.^10^ The change in biomechanical properties of trabecular bone^9^ might alter and increase the stress in other compartments, as in cortical bone in the hip, potentially increasing the risk for hip fractures. In addition, the effect of decreasing GH and IGF‐1 following treatment of cortical structures in acromegaly is less known; a reduction in cortical thickness could further compromise bone structural integrity. Indeed, mechanical and radiographic studies indicate that reduced cortical thickness increases the risk of hip fractures.^12^, ^15^, ^16^

Hip‐structure analysis (HSA) gives a DXA‐based estimation of bone geometry and structure in the total femur region.^17^, ^18^, ^19^, ^20^ The assessment of cortical thickness at the femoral neck, calcar, and proximal shaft can be derived from HSA and have been validated by pQCT measurements.^21^ Among the DXA‐HSA–derived parameters, increased hip axis length (HAL) and decreased cortical thickness were associated with an increased risk of hip fractures.^22^, ^23^

We hypothesized femoral cortical thickness would be increased in active acromegaly and decline following treatment. We therefore evaluated CBT using DXA‐HSA in active acromegaly compared with a control group of patients operated on for nonfunctioning pituitary adenomas (NFPAs) before any treatment and 1 year after pituitary surgery.

## Patients and Methods

### Population, baseline characteristics, and treatment

In this study we included 56 patients (33 males, 23 females) diagnosed with active acromegaly and 47 patients (20 males, 27 females) diagnosed with NFPA.

The patients with acromegaly were consecutively included at Oslo University Hospital Rikshospitalet, Norway, between 2005 and 2015. The diagnosis was based on clinical symptoms and confirmed by an elevated, age‐adjusted IGF‐1 level and failure to suppress GH by an oral glucose tolerance test. In this study, all patients had an DXA scan at baseline and 1 year after transsphenoidal pituitary surgery (1‐year PO).

The patients with NFPA were consecutively included at the same hospital between 2014 and 2017. The diagnosis of NFPA was based on an MR image of pituitary adenoma and lack of clinical manifestations from hypersecretion of pituitary hormones. A DXA scan of the NFPA patients was performed as part of routine follow‐up at baseline and 1‐year PO.

The definition of hypogonadism was as described previously.^10^ Accordingly, 8 female patients and 1 male patient with acromegaly and 20 female and 5 male patients with NFPA were considered hypogonadal; the other patients were defined as eugonadal.

### Ethics

Written informed consent was obtained from all participants and the study was conducted according to the Declaration of Helsinki II. The study was approved by the independent ethical committees in Norway (Regional Ethical Committee Health Region South‐East).

### Biochemical analysis

Plasma GH was measured by immunoassay (IMMULITE 2000; Siemens Healthcare GmbH, Erlangen, Germany) until 2015, then Roche Modular E170 between 2015 and 2016, and since 2017 a Roche Cobas e602 has been used (Roche Diagnostics, Basel, Switzerland).^24^ Sex steroids, sex hormone binding globuline (SHBG), luteinizing hormone, follicle‐stimulating hormone, and cortisol were assayed in plasma by Roche Modular E170 until 2016, then by the Roche Cobas e602 platform.

Serum IGF‐1 was analyzed by immunoassay (IMMULITE 2000; Siemens Healthcare GmbH) throughout the study period.^24^ All analyses were performed consecutively by accredited methods at the Department of Medical Biochemistry, Oslo University Hospital, according to standard protocols for the analytical methods. Intra‐ and interassay coefficients of variation were <5% for all assays.

### DXA measurements

BMD and total body composition were determined using DXA. A narrow fan beam (GE Healthcare Lunar Prodigy, Madison, WI, USA) densitometer was used; all scans were analyzed using software version 16 from GE Healthcare. No hardware changes were made during the study period. We analyzed anterior–posterior lumbar spine (LS; L_1_–L_4_), bilateral proximal femur, dual total hip, and dual femoral neck and presented BMD for all these regions. Further details on calculating BMD LS have been described previously.^10^ Absolute BMD values (g/cm^2^) and *Z*‐scores were estimated by comparison to the reference population present in the software, suitable for clinical use in the Norwegian population.^25^ Daily calibration was performed.^26^ The short‐ and long‐term coefficients of variation for our densitometer were 0.8% and 1.4%, respectively.^27^

### Assessment of trabecular bone score and hip structure analysis

**Figure 1:**
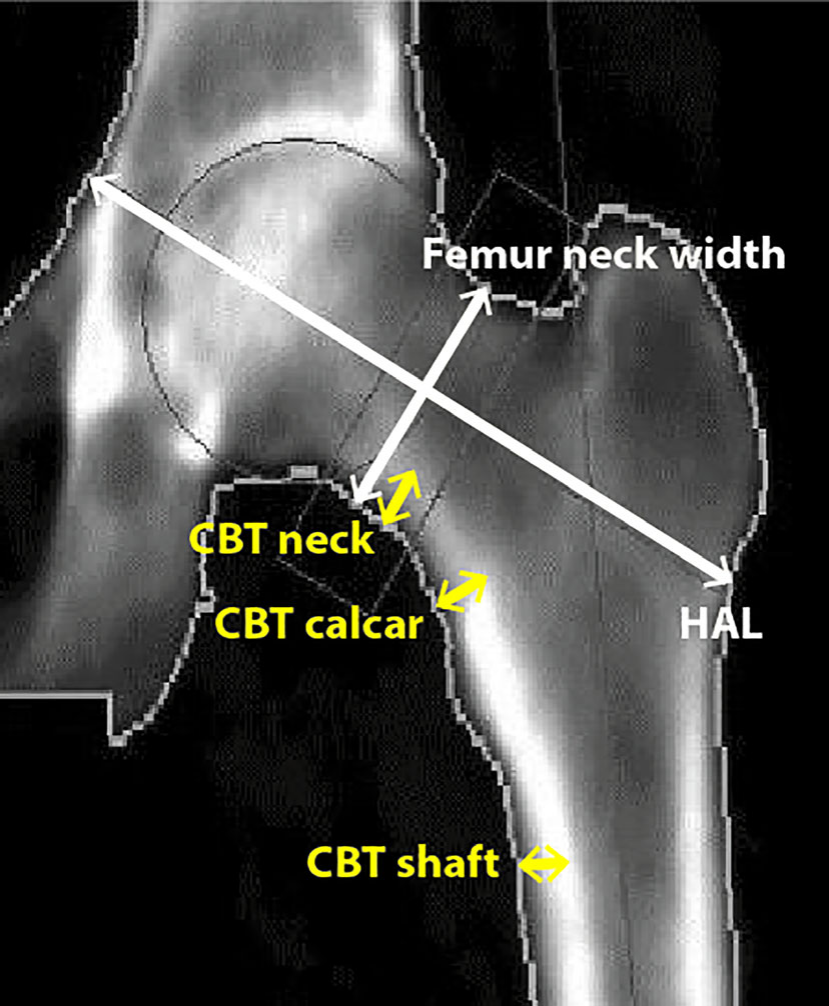
The DXA analysis program evaluated three regions of the proximal femur and derived cortical bone thickness (CBT) parameters for neck (CBT neck), calcar (CBT calcar), and shaft (CBT shaft), femur neck width, and hip axis length (HAL). Neck: CBT neck = the narrow neck across the narrowest point of the femoral neck; femur neck width = the femur neck width at the most narrow point; Calcar = CBT calcar at the intertrochanteric across the bisector of the neck. Shaft = CBT shaft at a define distance from the middle of the trochanter minor. Note: Here, all cursors were manually drawn for illustration only.

Lumbar spine TBS parameters were extracted from DXA L_1_–L_4_ images by using TBS iNsight software (version 2.1.2.0; Medimaps Group, Geneva, Switzerland), as previously described.^10^ All analyses were carried out by an International Society for Clinical Densitometry‐ (ISCD‐) certified densitometry technologist (KG). Compressed vertebras were excluded from TBS analyses. The principle for the HSA analyses (see Fig. 1) has been previously described in detail.^28^ In short, the HSA program uses bone mineral mass and dimensional data to assess the structural geometry of the proximal femur from DXA‐derived images of the hip as originally reported by Martin and Burr.^29^ Lines of pixel value pass over the bone axis in a bone mass image, and an HSA program presents fully mineralized adult cortical bone. The CBT was measured at three region sites of the proximal femur: (1) narrow neck (CBTneck); (2) intertrochanteric calcar (CBTcalcar); and (3) shaft (CBTshaft; Fig. 1). Cortical ratio is the ratio between cortical thickness and the diameter of the bone at the given site in %. Further, the program analyzes the femur neck width at the most narrow point (FW) and HAL. The HAL is defined as the distance from the greater trochanter to the inner pelvic rim.^30^ All measurement sites are automatically defined by the program.

HSA was performed on both hips, and the mean values calculated for each patient. In five patients, the automated HSA indicated HSA value error caused by positioning errors or artifacts. In these cases, only data from the one correctly assessed side were used.

### Statistics

Statistical analyses were conducted using SPSS for Windows, version 21.0 (SPSS, Chicago, IL, USA). In general, data are expressed as mean ± SD when normally distributed and median (25th, 75th percentile) when skewed. Comparison between the groups of acromegaly and NFPA was performed using a *t* test or Mann–Whitney *U* depending on distribution, and a chi‐square test for categorical variables. Associations between cortical bone variables and clinical markers were evaluated by Spearman correlation. Differences in the temporal course of cortical bone markers from baseline to 1‐year PO between acromegaly and NFPA were evaluated with repeated measures ANOVA à priori on log‐transformed variables, and with Bonferroni‐adjusted *t* tests between groups posteriori. Data from this analysis are expressed as estimated marginal means and 95% CIs. The groups (acromegaly and NFPA) were adjusted for age, sex, and hypogonadal status. Two‐tailed *p* values <0.05 were considered significant, except for interactions analysis where *p* < 0.1 was considered significant.

## Results

### Patient characteristics

The descriptive characteristics for the entire cohort of acromegaly (*n* = 56) and NFPA (*n* = 47) patients, DXA, and HSA data at baseline and 1‐year PO are presented in Table 1. Cross‐sectional analyses showed significant differences between patients classified as acromegaly and NFPA regarding hypogonadal status, age, and height, whereas no differences in BMI or weight between the groups at baseline or after 1‐year PO were found. Corresponding to disease activity, the patients with acromegaly had higher GH and IGF‐1 at baseline compared with NFPA patients. In patients with acromegaly, GH levels decreased from 8.2 (4.5, 22.9) μg/L to 1.0 (0.5, 2.8) μg/L and IGF‐1 from 113 (86, 139) nmol/L to 30^23^, ^31^ nmol/L (all *p* < 0.001) from baseline to 1‐year PO, as expected. Thirty patients had IGF‐1 ≤ upper limit of normal, and 40 had random GH ≤2.5 μg/L. Twenty‐three patients were on treatment with somatostatin analogues 1‐year PO as they did not achieve disease control after surgery alone. No patient in any group was on GH treatment during the study period. The patients with NFPA had low (normal) GH and IGF‐1 at baseline and no significant changes at 1‐year PO. Corticotrope pituitary deficiency was treated with physiological substitution doses of hydrocortisone (12.5 to 37.5 mg/d) in 3 patients with NFPA and 4 with acromegaly. Thyroxin treatment was given in 8 and 7 patients, respectively.

**Table 1:** Measurements at Baseline and 1‐Year Postoperative Follow‐Up of 56 Patients With Acromegaly and 47 Patients With Clinically Nonfunctioning Pituitary Adenomas

	Acromegaly		Change within group	NFPA		Change within group	Change between groups
	Baseline	1‐year PO		Baseline	1‐year PO		
Men/women (*n*)	33/23			20/27			
Somatostatin treatment (men/women) (*n*)		13/12					
Hypogonadal (men/women) (*n*)	1/8	1/9		5/20*	5/21**		
Age (years)	47.0 (13.3)	48.9 (13.4)	*p* < 0.001	59.4 (15.2)*	60.4 (15.1)**	*p* < 0.001	*p* < 0.001
Weight (kg)	88.3 (15.7)	89.0 (16.9)		83.7 (17.9)	85.0 (18.7)		
Height (m)	1.76 (0.10)	1.76 (0.10)		1.71 (0.09)*	1.70 (0.09)**	*p* = 0.019	
BMI (kg/m^2^)	28.4 (4.1)	28.5 (4.6)		28.7 (5.6)	29.2 (5.7)		
Disease activity							
GH (μg/L)	8.2 (4.5, 22.9)	1.0 (0.5, 2.8)	*p* < 0.001	0.2 (0.1, 0.7)*	0.6 (0.1, 0.8)**		*p* < 0.001
IGF‐1 (nmol/L)	113 (86, 139)	30 (23, 41)	*p* < 0.001	15 (9, 19)*	11 (8, 17)**	*p* = 0.009	*p* < 0.001
GF‐1/ULN	2.9 (1.9, 3.6)	0.9 (0.7, 1.3)	*p* < 0.001	0.5 (0.3, 0.6)*	0.5 (0.3, 0.6)**		*p* < 0.001
DXA							
LS; L1–L4 BMD (g/cm^2^)	1.21 (0.19)	1.25 (0.20)	*p* < 0.001	1.15 (0.18)	1.15 (0.18)		*p* = 0.001
LS; L1–L4 *Z*‐score	−0.25 (1.46)	0.06 (1.46)	*p* < 0.001	−0.18 (1.40)	−0.18 (1.48)		*p* < 0.001
TBS LS; L1–L4	1.320 (0.166)	1.299 (0.167)		1.257 (0.182)	1.256 (0.158)		
Dual femoral neck BMD (g/cm^2^)	1.02 (0.16)	1.02 (0.16)		0.89 (0.13)*	0.88 (0.13)**	*p* = 0.012	*p* = 0.021
Dual femoral neck *Z*‐score	0.17 (1.06)	0.31 (1.14)	*p* < 0.001	−0.37 (0.94)*	−0.43 (0.96)**		
Dual total hip BMD (g/cm^2^)	1.07 (0.16)	1.10 (0.15)		0.95 (0.14)*	0.94 (0.14)**	*p* = 0.028	*p* = 0.002
Dual total hip *Z*‐score	0.22 (1.10)	0.42 (1.11)	*p* < 0.001	−0.26 (1.04)*	−0.29 (1.04)#		
Hip structural analysis							
CBTneck (mm)	6.2 (4.7, 8.0)	5.6 (4.4, 6.7)	*p* = 0.004	5.1 (4.1, 6.4)*	5.5 (4.0, 6.7)		*p* = 0.002
Cortical ratio neck (%)	18.8 (14.2, 23.2)	16.1 (11.7, 19.7)	*p* = 0.003	15.8 (12.4, 18.7)*	16.0 (12.6, 19.3)		*p* = 0.003
CBTcalcar (mm)	4.8 (4.2, 5.7)	4.7 (4.1, 5.6)		4.0 (3.2, 4.5)*	4.3 (3.6, 5.0)	*p* = 0.001	*p* = 0.003
Cortical ratio calcar (%)	8.3 (6.5, 9.5)	7.8 (6.6, 9.4)		7.0 (6.0, 7.6)*	7.2 (6.5, 8.5)	*p* = 0.001	*p* = 0.003
CBTshaft (mm)	6.2 (5.1, 7.2)	6.0 (5.4, 6.9)		5.2 (4.6, 6.0)*	5.1 (4.2, 6.0)**		
Cortical ratio shaft (%)	18.7 (16.7, 21.0)	18.5 (16.6, 21.4)		17.0 (14.3, 19.8)*	16.8 (13.3, 20.2)**		
Min neck width (mm)	34.5 (31.8, 36.6)	34.7 (32.0, 37.0)	*p* = 0.004	32.4 (29.7, 35.3)*	32.3 (29.8.35.8)**		*p* = 0.040
Hip axis length (mm)	121 (108, 129)	123 (109, 131)		113 (104, 122)*	112 (104, 124)**		*p* = 0.025

Data are given as mean ± SD when normal distributed and median (25th, 75th) when skewed distributed.

PO = Postoperatively; NFPA = nonfunctioning pituitary adenoma; CBT = cortical bone thickness; GH = growth hormone; TBS = trabecular bone score; LS = lumbar spine; ULN = upper limit of normal.

* *p* < 0.05 between acromegaly and NFPA at baseline.

** *p* < 0.05 between acromegaly and NFPA at 1‐year PO.

Antiresorptive treatment with oral bisphosphonates was given to 3 patients with NFPA and 2 with acromegaly.

### DXA

As shown in Table 1, BMD and *Z*‐scores for femoral neck and total hip were significantly higher in patients with acromegaly compared with NFPA patients both at baseline and at 1‐year PO, whereas no difference in BMD or *Z*‐scores in LS or TBS was found between the groups. Further, we found an increase in BMD LS and improved *Z*‐scores in LS, femoral neck, and total hip for patients with acromegaly from baseline to 1‐year PO compared with a decrease in BMD femoral neck and BMD total hip in NFPA patients at the same time.

### Hip structure analysis

**Figure 2:**
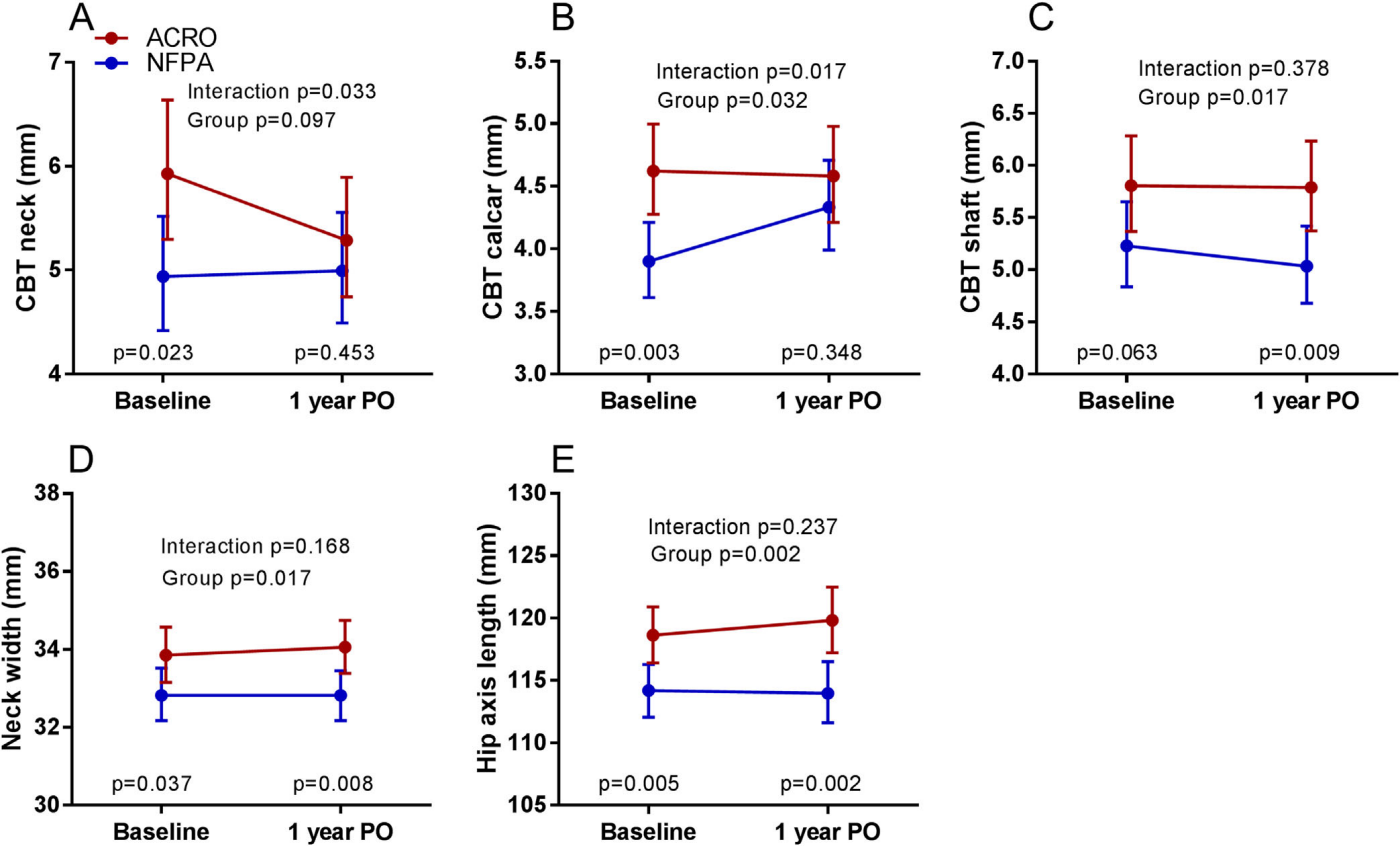
Hip structure analysis (HSA) at baseline and 1‐year postoperatively (PO) in acromegaly (ACRO, red line) and nonfunctioning pituitary adenoma (NFPA, blue line) patients. The interaction (group (ACRO, NFPA) * time) and the total group effect is given with Bonferroni‐adjusted *t* tests between groups at baseline and 1‐year PO. Data are adjusted for age, sex, and hypogonadal status and given as geometric mean and 95% CI.

We next investigated the cortical structure from baseline to 1‐year PO between the acromegaly and NFPA group, adjusting for sex, age, and hypogonadal status. As shown in Fig. 2, cortical thickness in the neck and calcar were higher at baseline in acromegaly, but these dimensions were comparable to the NFPA group at 1‐year PO because of a decrease in cortical thickness in the neck in acromegaly and an increase in thickness of the calcar in NFPA. No changes in cortical thickness of the shaft, FW, or HAL were observed; these indices remained higher at 1‐year PO in acromegaly.

### Associations between clinical data and cortical dimensions

**Figure 3:**
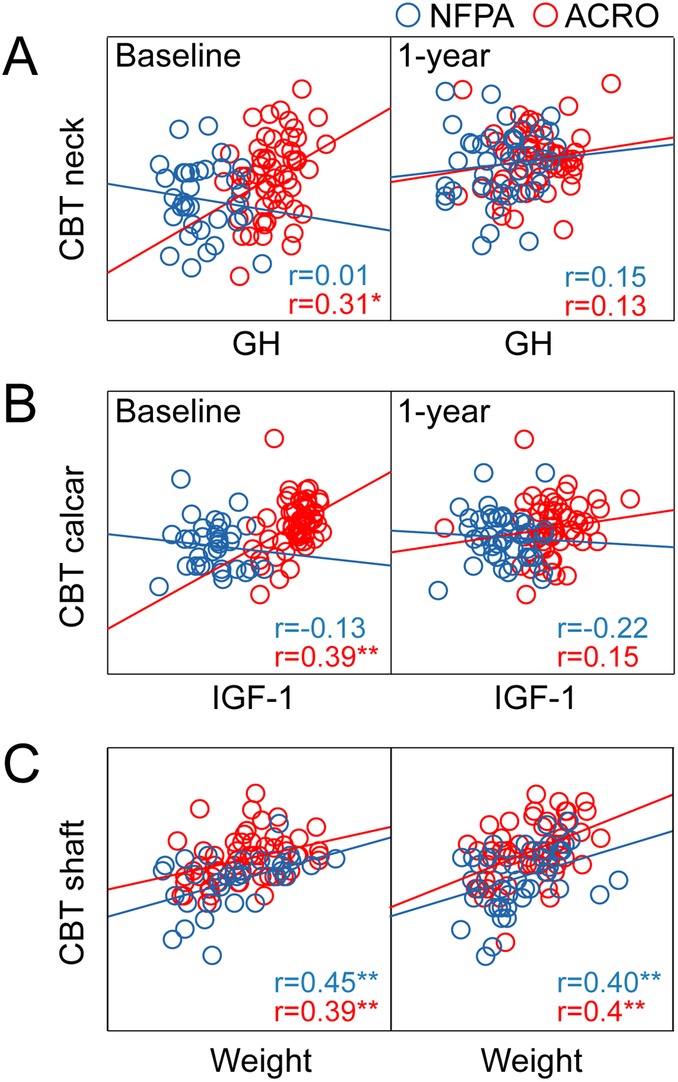
Correlations between cortical bone thickness (CBT) and clinical variables at baseline and 1 year postoperatively. (*A*) CBTneck and GH. (*B*) CBTcalcar and IGF‐1. (*C*) CBTshaft and weight. CBT = cortical bone thickness; GH = growth hormone; ACRO (red) = acromegaly; NFPA (blue) = nonfunctioning pituitary adenoma; GH = growth hormone; IGF‐1 = insulin‐like growth factor 1.

Important associations are depicted in Fig. 3, and an overview of all associations are presented in Table 2.

Focusing on cortical thickness, CBTneck correlated with GH levels and CBTcalcar with IGF‐1 levels at baseline in acromegaly, but not in NFPA, whereas this association was absent at 1 year in both groups.

As expected, weight was associated with CBT at all locations in NFPA, whereas CBTneck in acromegaly only was significantly associated with GH.

For FW and HAL, we found similar associations in both groups and at both time points regarding sex, age, height, and weight, whereas only IGF‐1 was associated with HAL at 1‐year PO in acromegaly.

## Discussion

In the present study, hip geometry and CBT was assessed by HSA in consecutively recruited patients with acromegaly and NFPA before treatment and 1 year after pituitary surgery. Our main findings in acromegaly compared with NFPA were: (1) increased CBT in the proximal femur in patients with active disease; (2) a decline in CBT in the femoral neck following treatment; (3) enhanced femoral hip dimensions that were unmodified after treatment; and (4) interactions between GH and IGF‐1 levels and cortical thickness in patients with active disease that were not present at follow‐up.

Our findings of increased femoral hip dimension and cortical thickness in patients with active acromegaly compared with NFPA support previous cross‐sectional studies using different methods for cortical bone assessment, and are in line with a specific effect of GH excess on bone geometry and cortical architecture.^32^, ^33^, ^34^ As expected, weight correlated with cortical thickness in both groups and measured sites, except for the femoral neck in acromegaly. In this site and group, only GH correlated with CBT. This was in contrast to calcar and shaft, where both IGF‐1 and weight were strongly correlated to CBT. This indicates that weight, IGF‐1, and GH differentially affect the femoral sites in active acromegaly, possibly related to different mechanical demands. Shear strain distribution changes from proximal to distal parts of the femur 35 and GH influences loading‐related bone formation in a permissive manner, modulating the responsiveness of bone tissue to mechanical stimuli by changing thresholds for bone formation.^36^ According to the results in the present study, CBTneck is the site most dependent on GH activity.

Surprisingly, the enlarged hip dimensions (FW and HAL) in active acromegaly were not correlated with GH or IGF‐1 levels. This agrees with the fact that dimensions were unmodified after pituitary surgery and remained elevated in acromegaly with similar correlations with anthropometric data as NFPA, before and after surgery. Similarly, the lack of change in cortical thickness of the shaft and correlation with weight reinforces that this site may be more influenced by mechanical forces than GH excess. In contrast, a decline in cortical thickness in the neck was observed in acromegaly following GH‐lowering treatment, and CBTneck and CBTcalcar were no longer correlated with GH and IGF‐1 1‐year PO. Periosteal bone formation seems to be more pronounced compared with the endocortical resorption in active acromegaly, resulting in an enlarged bone diameter with increased cortical thickness of potential importance for bone strength.^6^ Thus, the decline in cortical thickness after treatment could reflect enhanced endocortical bone resorption, as observed in normal aging.^37^, ^38^ Although cortical thickness was comparable to NFPA after treatment, the combination of decreased thickness and unchanged dimensions may result in inferior mechanical properties.

A higher risk for vertebral fractures, despite control of GH/IGF‐1 levels, has been established in acromegaly.^39^ For cortical bone, this paradox can partly be explained by local production and function of IGFs and their binding proteins^5^, ^7^ as emphasized in a recent review.^40^ Importantly, the risk of fractures is also associated with hypogonadism.^41^ We have previously demonstrated that treatment of acromegaly reduces TBS.^10^ Although trabecular bone contributes relatively more to the stability in the vertebra than in the hip, decreasing cortical stability in combination with loss of trabecular integrity could contribute to persistently increased fracture risk following treatment in acromegaly.^39^ Simultaneous alterations of both cortical and trabecular alterations have been demonstrated in early histomorphologic studies.^31^ Bone turnover is increased in acromegaly; accordingly, cortical porosity has been found increased in patients with active disease and vertebral fractures,^42^, ^43^ partly explaining the discrepancy between bone mass and strength. An association between cortical thickness in the hip and vertebral fractures was recently demonstrated.^34^ Future studies should evaluate the association between cortical thickness in the proximal femur and nonvertebral fractures in acromegaly.

Surprisingly, we observed an increase in CBTcalcar in the NFPA group. Possible explanations may be gradually improved pituitary and physical function following pituitary surgery. However, the improved gonadotrope function was not detected by the binary classification of hypogonadism in the present study. As both groups underwent the same treatment course, they may have undergone the same hypothesized improvement, emphasizing the strength of the study design with a control group. The differential course of CBTcalcar in the NFPA versus acromegaly group is a finding in line with CBTneck.

The strengths of the present longitudinal study are the acquisition of DXA measurements within a prospective protocol and the large cohort sizes. The study design allowed assessing the effect of GH excess before diagnosis, and the effect of GH decline 1 year after pituitary surgery by comparing HSA in acromegaly with patients with NFPA. It is an obvious strength that the control group (NFPA) underwent a comparable clinical course of diagnostic work‐up, transsphenoidal surgery, and postoperative follow‐up. However, certain limitations have to be addressed. The two groups were not optimally matched, as there were differences in gender distribution, gonadal status, and age. Nevertheless, statistical correction for the differences at baseline did not change the outcomes. Another confounding factor was the potential use of antiresorptive treatment with bisphosphonates. But only 3 NFPA patients were on bone active treatment, and exclusion of patients on antiresorptive treatment did not change the overall results of HSA. Three‐dimensional CT‐based measurements of the bone are more accurate than DXA‐based image acquisition. Thus, DXA HSA has method‐specific limitations compared with CT‐based assessment.^19^ However, pQCT is more resource‐demanding and exposes patients to more ionizing radiation, whereas DXA is easy to perform with little resource demand and with negligible radiation exposure.^19^

Taken together, the present study verifies previous observations of increased CBT in active acromegaly. The reduction of CBT after treatment compared with patients with NFPA indicates that endocortical bone resorption can be a contributing factor to persistently increased fracture risk in acromegaly despite increased bone dimensions and disease control.

## Disclosures

All authors state that they have no conflicts of interest.
